# Cerebral Artery Diameter in Inbred Mice Varies as a Function of Strain

**DOI:** 10.3389/fnana.2018.00010

**Published:** 2018-02-20

**Authors:** Baogang Qian, Robert F. Rudy, Tianxi Cai, Rose Du

**Affiliations:** ^1^Department of Neurosurgery, Brigham and Women's Hospital, Boston, MA, United States; ^2^Harvard Medical School, Harvard University, Boston, MA, United States; ^3^Biostatistics, Harvard T. H. Chan School of Public Health, Boston, MA, United States

**Keywords:** cerebral artery, cerebral circulation, mouse strain, variant anatomy, vascular anatomy

## Abstract

Many strains of mice are utilized in mouse models of cerebrovascular diseases. Variations in vascular anatomy between these strains has been documented and may influence the phenotype in stroke models. To address inter-strain variations in the circle of Willis anatomy, the diameters of internal carotid, posterior communicating, anterior cerebral, and middle cerebral arteries in 144 mice from 32 inbred strains were measured. Arterial diameters were analyzed as a function of animal weight, age, and strain. Variations in the structure of the circle of Willis across strains were observed and noted. While right-sided anterior cerebral arteries were significantly greater in diameter than their left-sided counterparts across most strains, variations in arterial diameter are strain specific. Adult mouse weight was not found to be associated with arterial diameter across strains, suggesting that cerebral artery size is associated with strain independently of weight. This study demonstrates strain dependent variations in the murine circle of Willis, which should be taken into consideration when studying mouse models of cerebrovascular diseases.

## Introduction

Estimates suggest ischemic and hemorrhagic stroke each accounted for greater than three million deaths worldwide in 2013, making cerebrovascular disease the third leading cause of death in the world (Roth et al., 2015). Research into cerebrovascular disease relies heavily on relies heavily on animal models, particularly rodent models, as experimental systems (Casals et al., 2011). Mouse models have been used to study the effects of arterial occlusion (Hata et al., 1998; Orset et al., 2007), hemorrhagic stroke (Rynkowski et al., 2008; Bühler et al., 2014), intracranial aneurysms (Morimoto et al., 2002), and vascular etiologies of neurodegenerative disease (Nishio et al., 2010).

Differences in cerebrovascular anatomy among mouse strains have been previously reported at the level of the major cerebral vessels and also at the microvascular level (Ward et al., 1990; Barone et al., 1993; Maeda et al., 1998; Beckmann, 2000; Wellons et al., 2000; Zhang et al., 2010). Specifically, variations in the structure of the circle of Willis have been documented in different strains of mice (Ward et al., 1990; Barone et al., 1993; Beckmann, 2000). At the microvascular level, the number and diameters of collateral vessels between the anterior cerebral artery (ACA) and middle cerebral artery (MCA) have been shown to differ across strains (Maeda et al., 1998; Zhang et al., 2010). In addition, the susceptibility to ischemia and the percentage of total cortical surface area supplied by the MCA, ACA, and posterior cerebral artery (PCA) can differ significantly across mouse strains (Sheldon et al., 1998; Majid et al., 2000; Zhang et al., 2010; Du et al., 2015). Several strains have been utilized for murine stroke models (Hata et al., 1998; Orset et al., 2007; Zhang et al., 2010; Du et al., 2015). However, despite the evidence that cerebrovascular anatomy differs among mouse strains, reports on variations in the circle of Willis have been confined to a handful of strains. Given the widespread use of several mouse strains as model organisms in cerebrovascular disease and the potential for variations in cerebral artery diameter to influence infarct volume, there is a need for better understanding of the variations among strains.

Based on these previous findings, we hypothesized cerebral artery diameters would differ across mouse strains. Thus, the goal of this study was to assess and document variations in the circle of Willis in 32 inbred mouse strains. These 32 strains were chosen to provide a broad range of stroke susceptibility (Du et al., 2015).

## Materials and methods

### Animal care and use statement

All animal care, housing, and experiments in this study were approved by and conducted in accordance with the Partners Healthcare System Policy on Humane Care and Use of Laboratory Animals from the Institutional Animal Care and Use Committee of the Harvard Medical Area Standing Committee on Animals. The experiments have been reported following the ARRIVE guidelines.

### Vessel perfusion

One hundred and forty-four male mice from 32 inbred strains, at least 8 weeks of age, were obtained from the Jackson Laboratory (Table 1). As there may be differences between male and female mice, we have restricted our study to male mice. Perfusion with India ink was performed to visualize cerebral blood vessels as previously described (Ward et al., 1990; Kitagawa et al., 1998; McGirt et al., 2002; Xue et al., 2014; Du et al., 2015). Briefly, mice were anesthetized with ketamine [100 mg/kg, intraperitoneal (IP)], xylazine (6 mg/kg, IP), and acepromazine (2 mg/kg, IP) (Patterson Veterinary Supply, St. Paul, MO) prior to transthoracic cannulation of the left ventricle and opening of the right atrium. Warm phosphate buffered saline was then perfused for 1–2 min, followed by 10% formalin (Fisher Scientific, Pittsburgh, PA) for 3 min, and then a 5% gelatin (USB Corporation, Cleveland, OH)/saline solution with India ink (American Mastertech Scientific, Lodi, CA) at 55°C for 15 s (McGirt et al., 2002; Du et al., 2015). The phosphate buffered saline was kept warm to avoid large temperature fluctuations when the India ink perfusion is performed. The India ink solution is warmed to 55°C to keep the gelatin dissolved. All perfusates were delivered at pressures of 60–80 mm Hg in order to maintain consistency. By using India ink perfusion, the diameters reported in this study represent the inner diameter of the vessel rather than the outer diameter. While it is possible for the vessel wall thickness to vary across strains, it is the inner diameter that is the more relevant parameter with regards to cerebral perfusion. Weight and age at the time of sacrifice prior to perfusion were recorded and mouse brains were removed for imaging.

**Table 1:** Number and mean weight, age, and vessel measurements with standard deviation for each strain.

**Strain**	**# of Mice**	**Wt (gms)**	**Age (wks)**	**Right ICA**	**Right PCoA**	**Right M1**	**Right A1**	**Left ICA**	**Left PCoA**	**Left M1**	**Left A1**
All strains	144	31.95 ± 6.75	19.37 ± 6.68	145.55 ± 22.79	120.40 ± 20.54	110.71 ± 19.65	124.64 ± 22.16	138.82 ± 20.60	118.52 ± 18.24	114.50 ± 19.20	112.55 ± 20.92
Number missing data	–	3	2	1	4	0	2	4	2	0	2
129S1/SvImJ	4	27.38 ± 1.10	19.75 ± 0.50	128.45 ± 32.74	124.61 ± 14.03	97.51 ± 26.20	107.68 ± 25.64	138.75 ± 23.26	118.27 ± 12.72	102.71 ± 18.71	123.58 ± 23.02
A/J	4	24.38 ± 4.09	20.00 ± 1.83	155.05 ± 5.21	134.95 ± 5.40	125.81 ± 9.57	128.77 ± 14.13	150.83 ± 21.82	107.43 ± 14.78	126.73 ± 8.65	129.55 ± 23.61
AKR/J	7	28.67 ± 1.87	13.00 ± 1.53	145.07 ± 10.20	113.80 ± 13.17	115.31 ± 12.98	128.75 ± 12.36	136.94 ± 13.94	115.03 ± 8.46	120.61 ± 15.20	106.56 ± 13.36
BALB/cJ	5	27.93 ± 0.69	14.20 ± 1.10	154.43 ± 11.72	119.67 ± 19.52	119.30 ± 5.66	125.14 ± 11.59	147.65 ± 11.53	129.96 ± 18.86	126.02 ± 4.19	124.91 ± 2.24
BTBR T+ tf/J	4	37.45 ± 1.79	26.50 ± 0.58	151.91 ± 13.07	123.94 ± 18.07	123.74 ± 14.08	130.28 ± 13.41	152.79 ± 21.59	128.98 ± 15.36	121.33 ± 8.32	134.69 ± 16.14
BUB/BnJ	3	28.97 ± 1.29	19.00 ± 0.00	168.60 ± 11.96	138.03 ± 9.38	121.43 ± 7.48	126.58 ± 14.65	137.10 ± 31.42	149.05 ± 22.17	125.72 ± 0.81	118.78 ± 38.68
C3H/HeJ	4	31.68 ± 0.55	22.00 ± 0.82	168.18 ± 33.51	168.47 ± 18.19	130.28 ± 22.17	149.31 ± 23.46	142.82 ± 18.66	154.16 ± 23.53	142.99 ± 12.33	110.35 ± 18.27
C57BL/10J	5	32.15 ± 1.09	26.00 ± 0.00	142.97 ± 11.06	121.25 ± 15.50	110.12 ± 8.19	122.57 ± 7.14	133.57 ± 9.03	110.94 ± 10.27	110.62 ± 10.21	105.43 ± 8.52
C57BL/6J	3	33.50 ± 3.27	20.67 ± 2.89	121.29 ± 12.79	98.73 ± 13.65	93.89 ± 12.92	98.50 ± 13.73	122.56 ± 12.47	102.81 ± 20.82	107.73 ± 18.56	101.20 ± 10.21
C57BLKS/J	8	26.23 ± 1.57	13.50 ± 4.84	120.39 ± 13.51	105.93 ± 6.58	83.94 ± 19.03	101.58 ± 13.38	115.98 ± 18.11	101.61 ± 5.89	91.45 ± 16.54	95.44 ± 13.70
C57BR/cdJ	4	36.23 ± 5.33	19.50 ± 1.73	165.28 ± 12.85	105.50 ± 9.77	134.50 ± 11.30	134.40 ± 17.35	146.61 ± 5.50	121.70 ± 10.21	116.24 ± 16.24	115.98 ± 10.83
C57L/J	4	33.83 ± 1.36	30.75 ± 1.26	160.56 ± 16.23	114.41 ± 7.41	123.61 ± 15.80	133.99 ± 22.19	140.28 ± 24.79	128.70 ± 19.40	125.42 ± 17.04	105.98 ± 14.25
CBA/J	5	30.66 ± 6.14	19.00 ± 7.31	155.90 ± 2.25	122.86 ± 10.81	111.97 ± 5.07	117.98 ± 14.93	136.24 ± 6.34	119.85 ± 13.01	107.93 ± 1.84	96.35 ± 14.62
CE/J	4	45.95 ± 1.38	20.00 ± 0.00	156.15 ± 8.15	147.62 ± 6.39	129.95 ± 3.07	130.14 ± 8.13	162.95 ± 6.99	148.36 ± 9.14	128.23 ± 10.65	135.30 ± 17.62
DBA/1J	4	26.05 ± 1.64	15.75 ± 1.50	112.16 ± 13.85	114.02 ± 4.76	83.81 ± 3.19	113.95 ± 7.77	105.99 ± 19.54	106.74 ± 9.30	87.18 ± 12.66	91.91 ± 20.83
DBA/2J	5	29.66 ± 2.39	15.40 ± 1.95	113.23 ± 18.71	98.55 ± 4.91	81.07 ± 14.34	102.35 ± 22.44	112.67 ± 12.09	99.67 ± 7.74	84.25 ± 14.02	96.41 ± 7.16
FVB/NJ	5	30.16 ± 1.11	15.00 ± 2.83	150.96 ± 16.58	128.74 ± 15.61	112.69 ± 11.70	131.32 ± 22.19	139.40 ± 14.18	117.53 ± 11.81	110.64 ± 21.60	118.67 ± 14.93
I/LnJ	3	26.27 ± 0.93	24.00 ± 0.00	161.47 ± 9.30	119.18 ± 12.23	112.69 ± 7.36	141.58 ± 9.24	144.35 ± 5.06	123.04 ± 6.91	115.42 ± 1.12	120.51 ± 2.65
KK/HlJ	4	41.10 ± 1.53	19.75 ± 0.50	128.34 ± 21.70	103.53 ± 2.92	102.45 ± 9.93	105.81 ± 20.87	144.15 ± 9.91	105.91 ± 12.99	113.72 ± 8.09	115.41 ± 8.06
LG/J	4	44.17 ± 1.53	16.00 ± 0.00	179.14 ± 16.34	132.11 ± 10.73	125.21 ± 14.24	161.46 ± 27.56	158.36 ± 13.18	119.15 ± 14.82	136.63 ± 6.93	123.38 ± 23.02
LP/J	4	27.45 ± 1.75	26.05 ± 2.10	163.75 ± 16.49	125.64 ± 11.62	115.94 ± 7.75	141.08 ± 7.09	159.17 ± 12.37	133.28 ± 13.62	122.73 ± 9.04	126.06 ± 21.67
MA/MyJ	4	29.15 ± 2.05	26.00 ± 0.00	138.73 ± 28.80	110.50 ± 15.54	110.19 ± 29.47	113.21 ± 24.53	144.89 ± 34.01	106.36 ± 8.30	122.12 ± 27.71	130.66 ± 43.91
MRL/MpJ	7	41.91 ± 6.38	16.43 ± 8.20	142.85 ± 22.27	107.56 ± 8.85	115.16 ± 18.38	120.31 ± 25.39	133.42 ± 28.67	107.48 ± 12.57	114.95 ± 16.37	90.94 ± 16.54
NOD/ShiLtJ	4	29.95 ± 1.25	17.00 ± 1.15	170.41 ± 5.99	131.97 ± 4.78	124.41 ± 8.94	141.35 ± 15.75	159.43 ± 14.84	136.64 ± 11.49	123.22 ± 8.44	135.26 ± 15.05
NON/ShiLtJ	4	37.77 ± 0.40	17.00 ± 2.00	137.02 ± 19.72	124.64 ± 24.14	103.53 ± 7.61	118.44 ± 15.55	123.73 ± 19.59	115.88 ± 4.90	108.65 ± 9.70	100.10 ± 14.24
NZO/HlLtJ	3	49.77 ± 2.20	20.00 ± 1.73	144.52 ± 4.58	157.25 ± 31.99	103.58 ± 9.54	142.46 ± 13.38	126.53 ± 9.76	127.04 ± 17.35	115.40 ± 10.16	91.61 ± 10.21
NZW/LacJ	4	34.75 ± 0.90	12.25 ± 2.06	162.06 ± 11.05	116.22 ± 7.86	123.76 ± 8.71	137.99 ± 17.28	149.34 ± 18.42	118.25 ± 17.89	130.96 ± 6.77	127.80 ± 18.45
P/J	4	27.77 ± 0.93	23.75 ± 0.50	140.84 ± 23.18	130.00 ± 28.60	116.13 ± 8.49	138.83 ± 15.48	143.24 ± 10.86	119.31 ± 13.69	122.42 ± 14.14	129.61 ± 6.38
PL/J	3	20.50 ± 0.71	8.00 ± 0.00	125.97 ± 17.63	105.86 ± 2.30	103.02 ± 22.17	102.72 ± 25.28	127.81 ± 8.01	106.23 ± 8.83	109.05 ± 5.69	110.45 ± 9.65
RIIIS/J	3	26.30 ± 1.45	25.67 ± 0.58	131.28 ± 4.89	89.32 ± 27.15	74.25 ± 27.62	107.40 ± 19.14	119.63 ± 11.17	109.50 ± 19.75	78.59 ± 24.53	98.48 ± 18.17
SJL/J	4	30.20 ± 1.87	23.25 ± 0.50	131.96 ± 19.01	116.77 ± 7.21	94.74 ± 14.47	118.85 ± 6.07	124.73 ± 15.55	104.25 ± 16.22	96.78 ± 23.24	108.05 ± 28.17
SM/J	4	30.68 ± 1.46	34.75 ± 0.50	151.32 ± 22.43	153.33 ± 28.40	129.75 ± 11.16	146.58 ± 33.28	162.95 ± 7.97	136.52 ± 14.85	135.24 ± 8.53	126.85 ± 21.64
SWR/J	7	27.06 ± 4.48	15.71 ± 13.88	146.06 ± 21.76	112.66 ± 19.33	110.52 ± 9.17	116.12 ± 24.17	142.44 ± 14.76	115.60 ± 13.16	114.46 ± 15.74	112.17 ± 12.35

*All vessel measurements are in μm (mean ± standard deviation). Wt, weight; gms, grams; wks, weeks*.

### Vessel measurement

**Figure 1:**
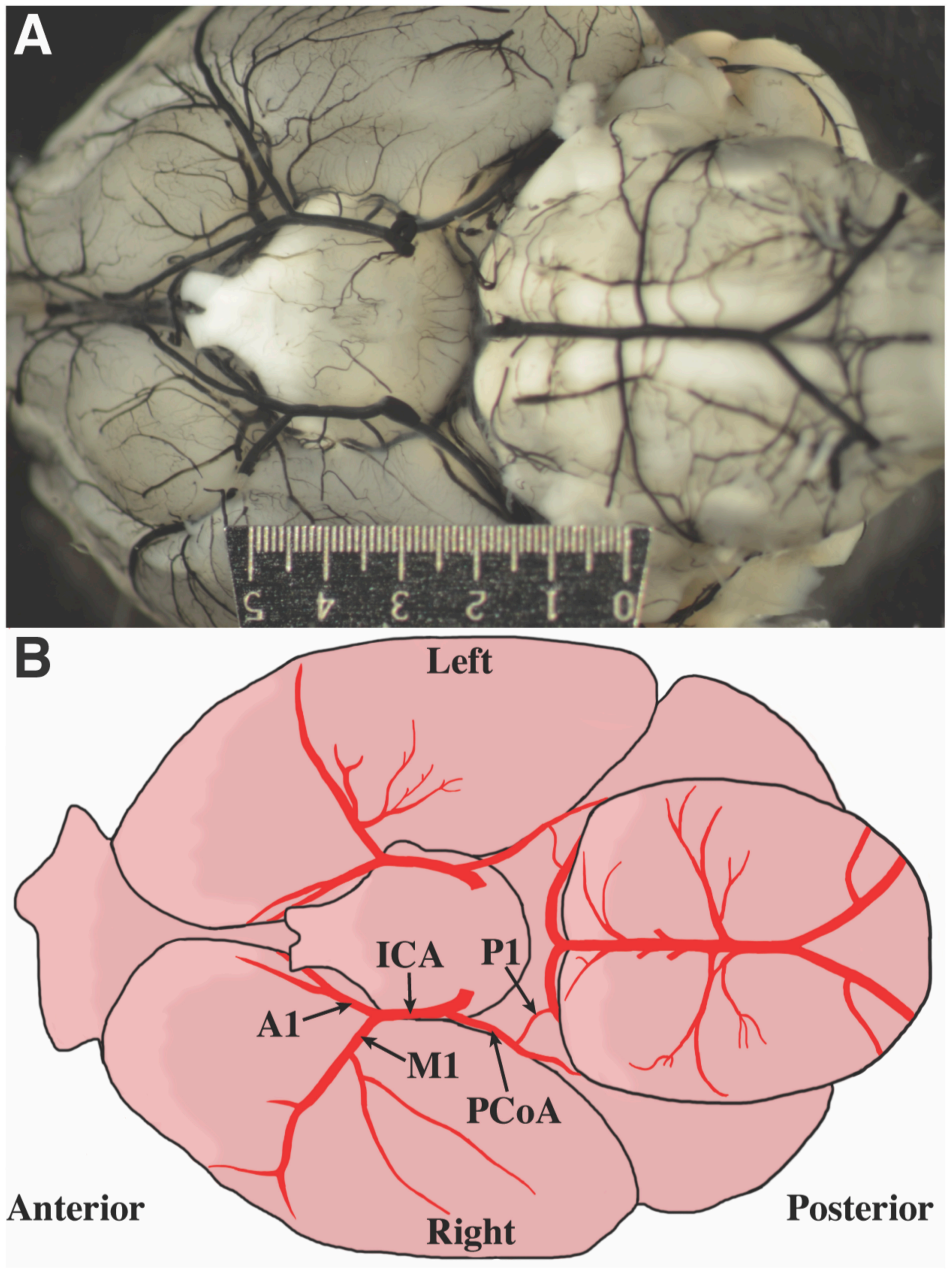
Defining mouse anatomic nomenclature based on human anatomy. **(A)** Representative photograph of inferior surface of the murine brain. The ruler measures 5 mm. **(B)** Illustration of the inferior surface of a murine brain. Blood vessel labels are shown for reference.

Photographs of the cerebral blood vessels perfused with India ink were obtained (Figure 1A). Standard human anatomical nomenclature was used to define murine artery names. An illustration of the vascular nomenclature used is shown in Figure 1B. Vessels were measured using ImageJ v1.47 (Schneider et al., 2012). Measurements, made by an investigator blinded to the age and weight of the mouse, were obtained from the right and left internal carotid artery (ICA), M1 segment of the MCA, A1 segment of the ACA, and posterior communicating artery (PCoA). To standardize the location at which vessel were measured, measurements were taken between 100 and 300 μm proximal (ICA diameter) and distal (M1 and A1 diameters) to the ICA bifurcation. PCoA measurements were taken proximal to the anastomosis with the first segment (P1) of the PCA, but due to overlapping tissues that may prevent good visualization, the measurements were taken 400–1200 μm from the PCoA take off. The presence of P1 on the image was noted. In many cases, although P1 segments were not observed, ruling them out entirely was not possible given overlapping tissue, thus the terms observed and not observed were used.

### Principal component analysis

Principal component analysis was then performed using data from the Mouse HapMap project (http://archive.broadinstitute.org/mouse/hapmap/). 132,285 Single nucleotide polymorphisms (SNPs) were extracted and those with a minor allele frequency ≤0.05 were excluded, as were SNPs missing in ≥10% of strains leaving a total of 105,258 included SNPs. Only mice with complete datasets were used and the mean diameter of all vessels in each mouse was used as the phenotype. Using Plink v1.9 (Purcell et al., 2007) (http://pngu.mgh.harvard.edu/purcell/plink/), principal component analysis was performed and the first two eigenvectors were plotted with *ggplot2* in R (Wickham, 2009). Clusters were then labeled with vessel diameters categorized into four groups (≥80–<100 μm, ≥100–<120 μm, ≥120–<140 μm, ≥140 μm).

### Heatmap

Given C57BL/6J mice are commonly used in stroke models and were utilized by Hata et al. (1998) to study the association between weight and the diameter of filament need to occlude the MCA, we normalized the mean arterial diameters by dividing the mean vessel diameter for each strain by the average diameter in the C57BL/6J mice. The strains were subsequently clustered using a hierarchical clustering method based on the Euclidean distance matrix and complete agglomeration (*gplots* package in R Warnes et al., 2016).

### Statistical analysis

Statistical analysis was conducted in R v3.4 (R Core Team, 2017). Only complete (non-missing) data was used for the analysis. Wilcoxon signed-rank tests and Spearman correlations were used to determine differences in the size of and correlations between ipsilateral and the corresponding contralateral arteries. ANOVA between multivariate linear regression models with vessel size as the dependent variable and weight, age, and strain as covariates were used to evaluate the effect of each covariate on vessel diameter. *P*-values were adjusted for multiple testing via the Benjamini-Yekutieli correction, which also accounts for correlation among the test statistics (Benjamini and Yekutieli, 2001). Adjusted *P*-values of less than 0.05 were considered significant. Additionally, the *plyr* (Wickham, 2011), *dplyr* (Wickham et al., 2017), *tidyr* (Wickham, 2017b), *stringr* (Wickham, 2017a), *broom* (Robinson, 2017), and *RColorBrewer* (Neuwirth, 2014) packages in R were utilized for data organization and figure generation.

## Results

### Vessel size

**Figure 2:**
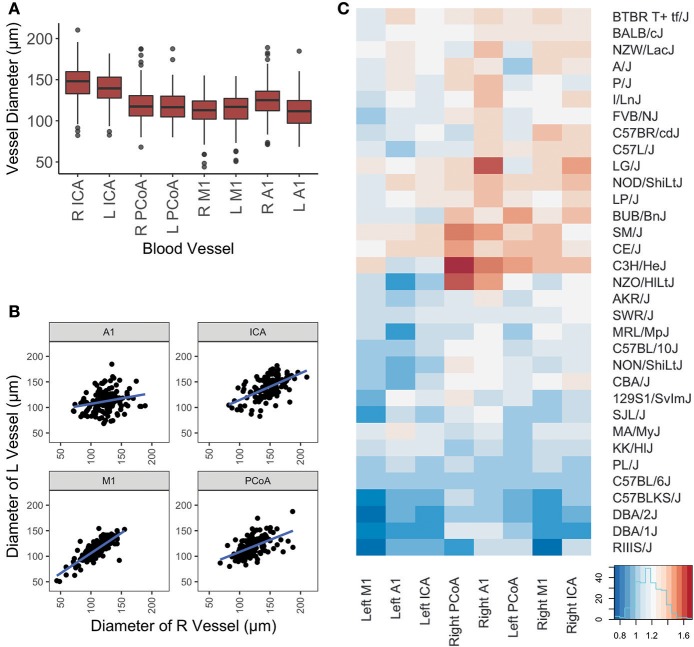
Graphical representation of vessel diameters. **(A)** Boxplot depicting distribution of measurements for each vessel. Box represents 1st quartile through 3rd quartile, with line indicating median. Whiskers extend no more than 1.5x the interquartile range. Data points beyond that are represented by a dot. **(B)** Scatterplot of left vs. right diameters for each vessel included in this study. Linear regression lines are depicted for each artery. **(C)** Graphical display of mouse vessel diameters by strain. Normalized mean vessel diameters were used to cluster strains, which are shown in a heatmap to provide visualization of the vascular patterns for each strain.

One hundred and forty-four male mice derived from 32 inbred strains were included in this study. Average measurements for all mice and for each strain separately are provided in Table 1 and distributions per vessel are shown in Figure 2A.

Differences in ipsilateral vessel diameters and left-sided vs. right-sided vessel diameters were examined (Table 2). Ipsilateral vessel comparisons were performed to determine relative sizes of vessels on each side, as a proxy for ascertaining the relative proportion of blood directed to the ACA, MCA, and posterior circulation from each ICA. ICAs were the largest vessel bilaterally. Right-sided A1 segments were larger than right-sided M1s, whereas on the left side these segments did not differ in size. Left PCoAs were larger than both left M1s and A1s, whereas right PCoAs were larger than right M1s but did not differ in size from right A1s. Right-sided A1s and ICAs were larger than their left-sided counterparts, whereas left-sided M1s were larger than those on the right. All four vessels measured demonstrated positive correlations between left and right, shown in Figure 2B, and Spearman correlation coefficient was statistically significant for all comparisons, with the exception of the right and left A1 segments (Supplementary Table 1).

**Table 2:** Comparisons between cerebral arteries with Benjamini-Yekutieli corrected *P*-values.

**Vessel 1**	**Vessel 2**	**Wilcoxon signed rank test estimate [95% confidence interval]**	***P*-value**	**Adjusted *P*-value**
R ICA	R M1	34.34 [31.91 to 36.87]	<**0.001**	<**0.001**
R ICA	R A1	21.10 [18.78 to 23.37]	<**0.001**	<**0.001**
R ICA	R PCoA	26.62 [22.82 to 30.22]	<**0.001**	<**0.001**
R M1	R A1	−13.34 [−16.54 to −10.36]	<**0.001**	<**0.001**
R M1	R PCoA	−9.70 [−12.98 to −6.44]	<**0.001**	<**0.001**
R A1	R PCoA	3.57 [−0.11 to 7.25]	0.055	1
L ICA	L M1	24.32 [21.96 to 26.70]	<**0.001**	<**0.001**
L ICA	L A1	25.54 [23.30 to 27.80]	<**0.001**	<**0.001**
L ICA	L PCoA	20.73 [17.37 to 23.97]	<**0.001**	<**0.001**
L M1	L A1	1.06 [−1.83 to 4.07]	0.466	1
L M1	L PCoA	−4.26 [−7.13 to −1.25]	**0.007**	0.135
L A1	L PCoA	−6.40 [−9.43 to −3.25]	<**0.001**	**0.003**
R ICA	L ICA	7.58 [4.01 to 11.02]	<**0.001**	<**0.001**
R PCoA	L PCoA	1.30 [−1.51 to 4.21]	0.384	1
R M1	L M1	−3.50 [−5.46 to −1.84]	<**0.001**	**0.003**
R A1	L A1	11.53 [6.86 to 16.04]	<**0.001**	<**0.001**

*P-values < 0.05 are bolded*.

We present in Figure 2C the heatmap of the mean arterial diameters for each strain normalized to those of C57BL/6J diameters. ANOVA comparing the multivariate models including weight, age, and strain and the models including only weight and age demonstrated that strain significantly contributes to the variation of vessel diameter at all arterial locations except for the left ICA and left A1 (Table 3). On the other hand, weight and age in adult mice were not significantly associated with vessel diameter at any location after accounting for strain (Table 3). Principal component analysis of the SNPs did not reveal clustering with respect to vessel diameters (Supplementary Figure 1).

**Table 3:** ANOVA of multivariate models including the explanatory variables strain, weight, and age compared to a multivariate model with one of those variables removed.

**Artery**	**Variable of interest**	**Raw *P*-value**	**Adjusted *P*-value**
Right ICA	Strain	<**0.001**	<**0.001**
Right PCoA	Strain	<**0.001**	<**0.001**
Right M1	Strain	<**0.001**	<**0.001**
Right A1	Strain	<**0.001**	**0.005**
Left ICA	Strain	<**0.001**	**0.003**
Left PCoA	Strain	<**0.001**	<**0.001**
Left M1	Strain	<**0.001**	<**0.001**
Left A1	Strain	<**0.001**	**0.011**
Right ICA	Weight	0.402	1
Right PCoA	Weight	0.763	1
Right M1	Weight	0.802	1
Right A1	Weight	0.787	1
Left ICA	Weight	0.992	1
Left PCoA	Weight	0.060	1
Left M1	Weight	0.720	1
Left A1	Weight	0.367	1
Right ICA	Age	0.258	1
Right PCoA	Age	0.087	1
Right M1	Age	0.642	1
Right A1	Age	**0.030**	0.853
Left ICA	Age	0.141	1
Left PCoA	Age	0.614	1
Left M1	Age	0.637	1
Left A1	Age	0.461	1

*Each row indicates a separate ANOVA for the respective vessel location. P-values were adjusted using the Benjamini-Yekutieli procedure. P-values < 0.05 are bolded*.

### Anatomical variants

**Figure 3:**
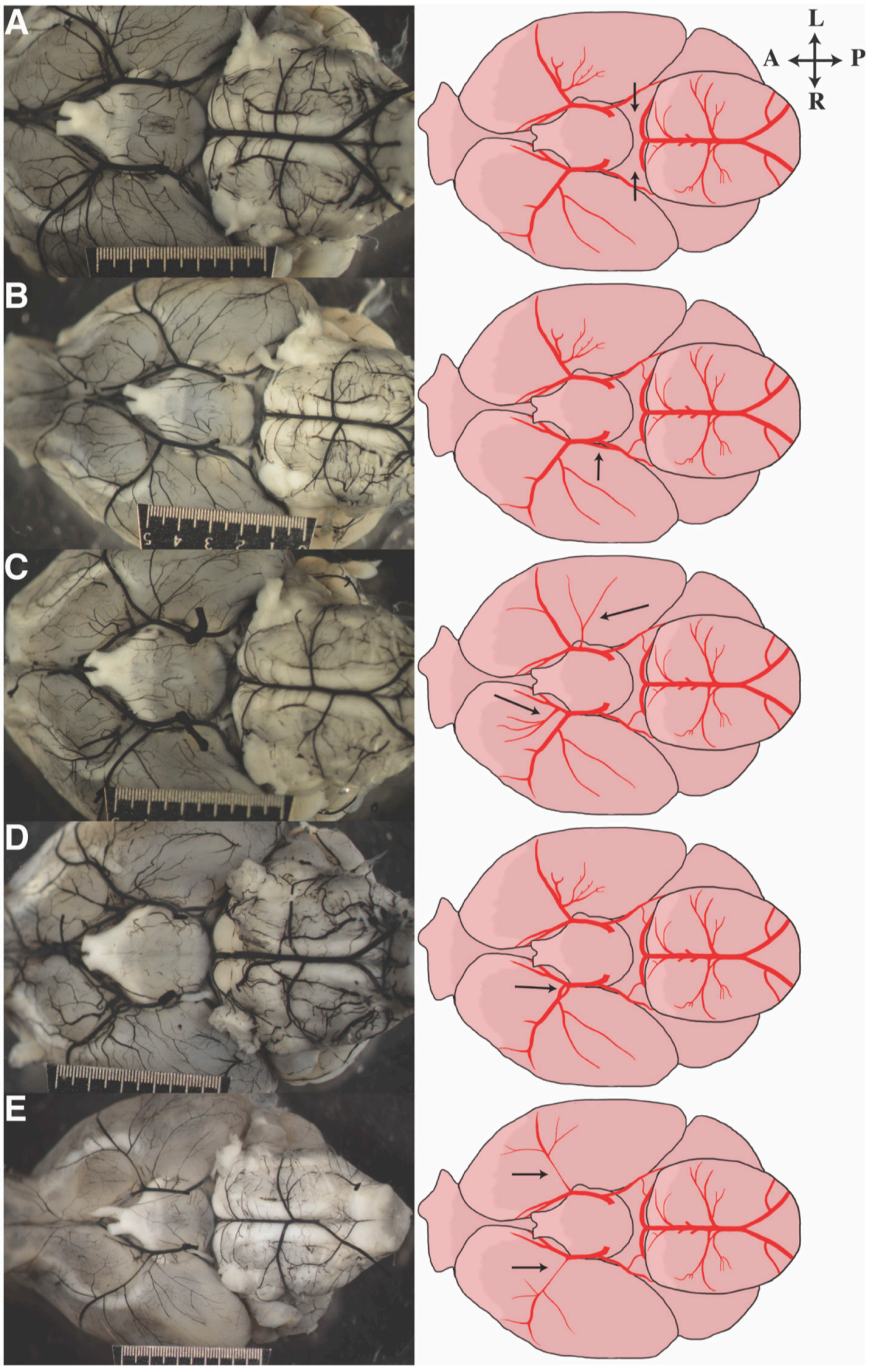
Representative images of anatomic variants observed in our cohort of mice. Photograph on the left corresponds to the illustration on the right. Arrows direct attention to the anatomic feature of interest: **(A)** bilateral aplastic P1s, **(B)** duplicated PCoA take off, **(C)** bilateral duplicated/accessory MCAs, **(D)** fenestrated M1, and **(E)** bilateral hypoplastic proximal MCAs.

Numerous anatomic variants were observed during this study. The most common anomaly observed was absent or unilateral P1s (Figure 3A, Supplementary Table 2). Duplicated PCoAs were also observed, with two PCoAs originating from one ICA in four mice [A/J, FVB/NJ, and SM/J (2 mice) strains] (Figure 3B). MCA variation was the next most common site of variation. Accessory MCAs, namely, arteries originating in the distal ICA and traversing the typical MCA territory were observed in several mice across multiple strains (C57BL/6J, C57L/J, CE/J, C57BR/cdJ, DBA/1J, FVB/NJ, MA/MyJ, NOD/ShiLtJ, NON/ShiLtJ, NZO/HlLtJ, PL/J, RIIIS/J, SWR/J) (Figure 3C). These additional MCA territory vessels varied in size from small to nearly identical to the ipsilateral MCA. Two mice from different strains (C57BR/cdJ, FVB/NJ) with fenestrated M1s were also observed (Figure 3D). Proximally narrow MCAs were also observed to varying degrees, with apparent distal dilatation (Figure 3E).

## Discussion

While mouse models are ubiquitous in cerebrovascular research, there has not been a study investigating variation in the size of murine cerebral arteries across a large number of inbred strains. In this study, we analyzed variations in the cerebral artery anatomy of 32 inbred mouse strains and found differences in vessel diameter based on both sidedness and strain.

### Nomenclature for mouse circle of willis

A consistent anatomic definition of murine circle of Willis anatomy is important for clarity and interpretation. However, cerebral artery naming has not been consistent in the mouse literature. In particular, when compared to human anatomical labeling (Zeal and Rhoton, 1978; Schomer et al., 1994), the definition of the PCoA and P1 are often reversed (Barone et al., 1993; Kitagawa et al., 1998; Beckmann, 2000). In humans, the PCoA is defined as the vessel connecting the ICA to the PCA whereas the PCA arises from the basilar artery. P1 is the PCA segment starting with the origin of PCA until the anastomosis with PCoA. To maintain consistency, we used a naming system based on human anatomic nomenclature in this study.

### Variations of cerebral arteries in different locations

Unsurprisingly, the ICA was found to be larger than other intracranial vessels. The right A1 segment was larger than the left A1 suggesting right A1 dominance. The ACA supplies the olfactory bulb, a robust structure in mice. In rats, asymmetry of the olfactory bulb has been reported, with the right side larger than the left (Heine and Galaburda, 1986). However, a study using MRI to investigate size asymmetry in mice did not find a statistically significant difference in the size of the right and left olfactory bulbs (Barbeito-Andrés et al., 2016). In addition to A1 asymmetry, we found the right ICA to be larger than the left ICA, and the left M1 to be larger than the M1. The MCA asymmetry may reflect the larger A1 on the left demanding more flow resulting in a smaller left M1, which would also explain the discrepancy in ipsilateral comparisons between the A1 and M1 on the right and left side. These data pose questions regarding the functional significance of arterial asymmetry, particularly that of A1.

### Variations of cerebral arteries across different inbred strains

Variations in diameters across strains in this study were not secondary to variations in weight, suggesting cerebral artery diameters are largely under genetic control in mice. The lack of clustering with mean vessel diameter in the principal component analysis should not be interpreted as evidence against predominant genetic control, but rather that vessel diameter phenotype is a complex phenotype.

These results appear to conflict with prior studies that found a significant, positive correlation between mouse weight and the filament diameter needed to produce an ischemic infarct (Hata et al., 1998). However, our study was limited to adult mice at least 8 weeks of age resulting in a small intrastrain variation in weight, whereas the study by Hata et al. utilized C57BL/6J mice (*N* = 35) with weights ranging from 18 to 33 g suggesting that some of these mice were younger in age than the ones included in this study. Large variations in weight, indicative of growing mice, likely do influence blood vessel diameters within a strain. However, strain, but not weight, was found to account for vessel variations in a multivariate model in adult mice. Thus, the dependence of vessel size on strain occurs independently of the mean weight of adult mice in that strain.

Zhang et al. reported differences in the percentage of brain surface perfused by large cerebral arteries across inbred mouse strains (Zhang et al., 2010). However, we found that some strains with relatively small areas supplied by the MCA according to Zhang et al. (e.g., NOD/ShiLtJ and NZW/LacJ) have large M1 diameters, suggesting that the size of the arterial territory may not be driven by the diameter of the vessel. While the diameter of arteries does not necessarily correspond to the percent of the brain surface area perfused, the study by Zhang et al. provides further evidence that variations in cerebrovascular anatomy are driven by strain.

Variation in diameters of major cerebral arteries amongst mouse strains has implications for occlusive ischemic models, including MCA occlusion via filament. The recent IMPROVE guidelines on appropriate utilization of *in vivo* ischemia modeling recommend using the smallest diameter filament that produces a reliable infarct (Percie du Sert et al., 2017). These data suggest that strain should be considered when choosing initial filament diameters when performing the MCA occlusion model.

### Variations in circle of willis anatomy

We observed several anatomic variants during the course of the study. Unilateral or bilateral absence of P1 was common. In our study, no BTBR T+ tf/J or RIIIS/J mice were found to have visible bilateral P1 segments, whereas A/J, AKR/J, C57BL/10J, CE/J, DBA/2J, and P/J mice all had observable bilateral P1 segments. Variations in P1 presence are significant for stroke research as PCA territory infarctions could occur with anterior circulation occlusion when there is an absence of P1. This may contribute to the variation in infarct size in an MCA occlusion model. Furthermore, the absence of P1 segments results in a non-intact circle of Willis which is known to increase the risk of hypotensive infarcts in humans (Schomer et al., 1994). MCA variation has been documented previously in over 17% of Sprague-Dawley rats (Fox et al., 1993). Duplicated and accessory MCAs have also been reported in humans, some of which arose from the proximal ACA and others from the distal ICA (Komiyama et al., 1997, 1998; Chang and Kim, 2011; Liu et al., 2015). Case reports of human patients with accessory MCAs and MCA occlusion report favorable outcomes, suggesting these vessels can perfuse at least some of the at-risk territory following MCA occlusion (Komiyama et al., 1997; Liu et al., 2015). Thus, it is plausible that in mice, duplicated MCAs could potentially lead to smaller infarct volumes following MCAO.

### Study limitations

This study has important limitations. A total of 144 mice were included, with as few as three mice per strain, limiting the generalizability of these results. We can thus draw conclusions regarding variations in vessel diameters across strain but not within strains. The precision of the measurements is limited by potential subtle variations in India ink perfusion and by image resolution. To maximize reproducibility, we have performed the India ink perfusion using heated ink and perfused under pressures of 60–80 mm Hg. An adequate view of the P1 segments was often limited as these segments were sometimes hidden from view near the junction of the pons and midbrain. Thus, we are unable to rule out the presence of P1 segments. These data do not demonstrate any functional differences; rather they document variations in the diameters of these vessels.

In summary, this study investigated anatomic differences in the caliber of cerebral arteries between 32 inbred strains of mice, taking advantage of a large number of strains to evaluate the impact of strain on artery size, which has not been done to this extent. These data suggest mouse cerebral artery diameter is determined by genetics rather than the mean size of the adult mouse, as evidenced by variations due to strain and not weight. Thus, data from one strain should be interpreted with caution when compared to mice from different strains. To increase the robustness of these data, a larger number of mice per strain will be required in future studies. Additional studies will also be necessary to determine the underlying genetic mechanisms driving cerebral artery diameters.

## Author contributions

BQ: Acquired data, critically revised the manuscript; RR: Acquired and analyzed data, drafted the manuscript; TC: Analyzed the data, critically revised the manuscript; RD: Conceived of and designed the study, critically revised the manuscript.

### Conflict of interest statement

The authors declare that the research was conducted in the absence of any commercial or financial relationships that could be construed as a potential conflict of interest.
