# Supplementary material for: Cerebral Artery Diameter in Inbred Mice Varies as a Function of Strain
Source: Front Neuroanat. 2018 Feb 20;12:10. doi: 10.3389/fnana.2018.00010 (PMC5826213; doi:10.3389/fnana.2018.00010)
Supplement: Supplementary file 1 [file DataSheet1.pdf]

frontiers

Supplementary Material

Cerebral Artery Diameter in Inbred Mice Varies as a Function of Strain

Baogang Qian[1,2,†], Robert F. Rudy[1,2,†], Tianxi Cai[3], Rose Du[1,2,*]

[1]Department of Neurosurgery, Brigham and Women's Hospital, Boston, MA, USA
[2]Harvard Medical School, Boston, MA, USA

[3]Biostatistics, Harvard T. H. Chan School of Public Health, Boston, MA

[†]These authors contributed equally to this work
*Corresponding Author

Address for Correspondence:
Rose Du, MD, PhD
Department of Neurosurgery
Brigham and Women's Hospital
75 Francis Street
Boston, MA 02115, USA
Email: rdu@bwh.harvard.edu
Phone: 617-732-6600
Fax: 617-734-8342

1 Supplementary Methods

Power Calculation

The *pwr* package in R was used to perform power calculations[1]. Assuming a difference in vessel diameters of 35 μm and a standard deviation of 10 μm, we have a power of 0.89 for n=3 per group for a significance level of 0.05.

2 Supplementary Figures and Tables

Supplementary Table 1. Spearman correlation between vessel 1 and vessel 2. The Benjamini-Yekutieli procedure was used to obtain adjusted P values. P values <0.05 are bolded.

Vessel 1	Vessel 2	Spearman coefficient	Raw P value	Adjusted P value
R ICA	R M1	0.72	**<0.001**	**<0.001**
R ICA	R A1	0.71	**<0.001**	**<0.001**
R ICA	R PCoA	0.52	**<0.001**	**<0.001**
R M1	R A1	0.58	**<0.001**	**<0.001**
R M1	R PCoA	0.50	**<0.001**	**<0.001**
R A1	R PCoA	0.49	**<0.001**	**<0.001**
L ICA	L M1	0.74	**<0.001**	**<0.001**
L ICA	L A1	0.75	**<0.001**	**<0.001**
L ICA	L PCoA	0.46	**<0.001**	**<0.001**
L M1	L A1	0.55	**<0.001**	**<0.001**
L M1	L PCoA	0.50	**<0.001**	**<0.001**
L A1	L PCoA	0.46	**<0.001**	**<0.001**
R ICA	L ICA	0.55	**<0.001**	**<0.001**
R PCoA	L PCoA	0.53	**<0.001**	**<0.001**
R M1	L M1	0.77	**<0.001**	**<0.001**
R A1	L A1	0.24	**0.004**	0.060

Supplementary Table 2. Number of mice per strain with observed P1 segment of posterior cerebral arteries.

Strain	R Unilateral P1	L Unilateral P1	Bilateral P1	No P1 Observed
129S1/SvImJ	0	2	2	0
A/J	0	0	4	0
AKR/J	0	0	7	0
BALB/cJ	2	1	1	1
BTBR T+ tf/J	0	1	0	3
BUB/BnJ	0	1	2	0
C3H/HeJ	1	1	2	0
C57BL/10J	0	0	5	0
C57BL/6J	0	0	1	2
C57BLKS/J	1	1	6	0
C57BR/cdJ	1	1	1	1
C57L/J	1	1	1	1
CBA/J	0	1	4	0
CE/J	0	0	4	0
DBA/1J	1	0	2	1
DBA/2J	0	0	5	0
FVB/NJ	0	0	4	1
I/LnJ	0	1	2	0
KK/HlJ	1	1	2	0
LG/J	0	1	2	1
LP/J	0	1	3	0
MA/MyJ	0	1	2	1
MRL/MpJ	2	1	0	4
NOD/ShiLtJ	0	1	2	1
NON/ShiLtJ	2	1	1	0
NZO/HlLtJ	1	0	2	0
NZW/LacJ	1	2	1	0
P/J	0	0	4	0
PL/J	1	0	2	0
RIIIS/J	1	0	0	2
SJL/J	2	0	2	0
SM/J	0	1	3	0
SWR/J	0	2	5	0

Supplementary Figure 1. First two components from a principal component analysis of SNPs across mouse strains. Mean vessel diameters across all vessels using only mice with complete vessel data are coded as follows: 0: ≥80 - <100 µm, 1: ≥100 - <120 µm, 2: ≥120 - <140 µm, and 3: ≥140 µm. Lines indicate where dataset lies on the plot, drawn to avoid overlap.

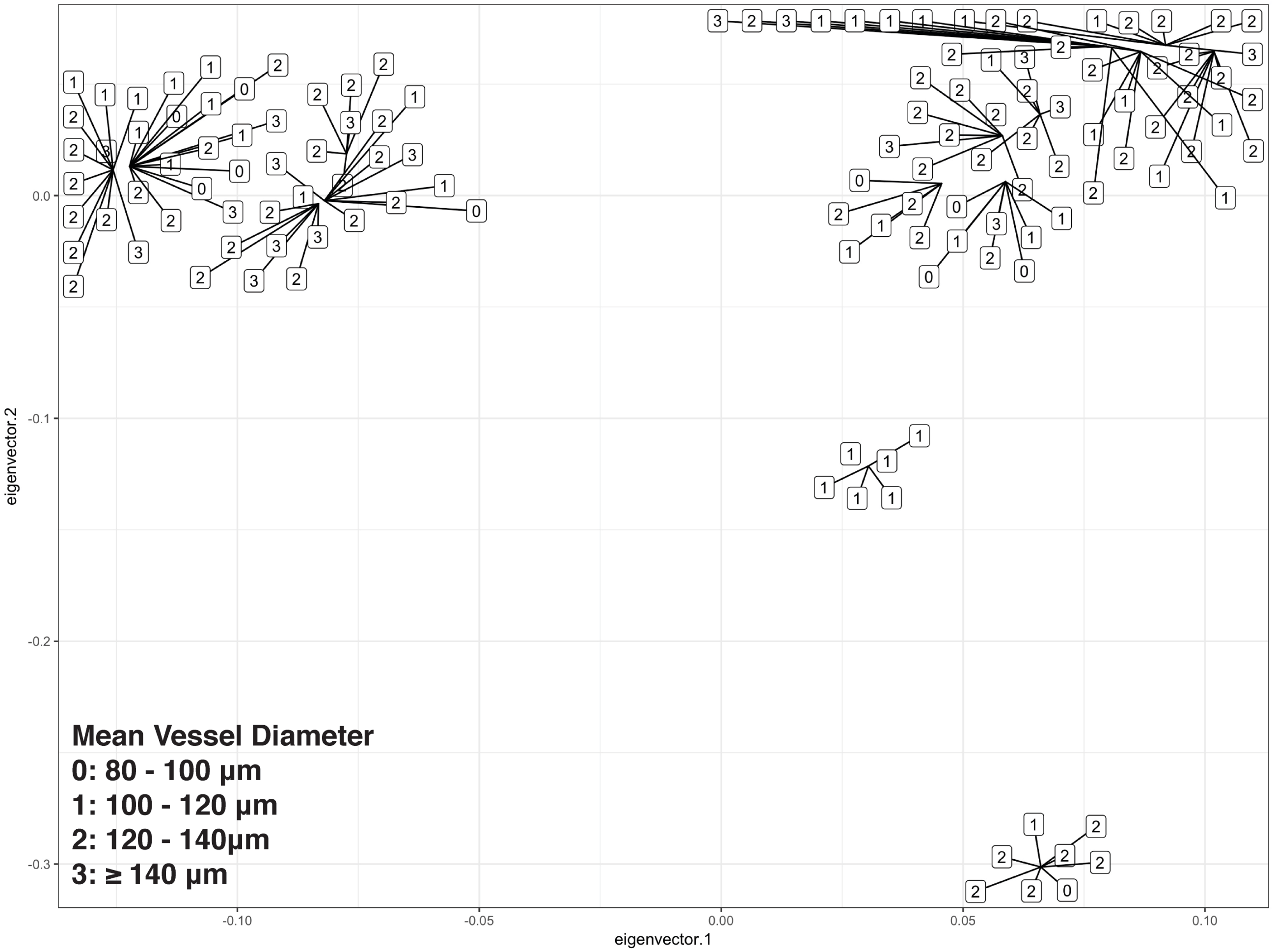

Supplementary Reference

1. Champely S. Pwr: Basic functions for power analysis, r package version 1.2-0. 2016
